# Caregiver burden and its sociodemographic determinants in family caregivers of patients with schizophrenia attending a psychiatric tertiary hospital in South Africa

**DOI:** 10.4102/sajpsychiatry.v30i0.2252

**Published:** 2024-06-21

**Authors:** Chioma O. Onyia, Julia S. Lethole, Gbenga Olorunfemi, Nnabuike C. Ngene

**Affiliations:** 1 Department of Psychiatry, School of Medicine, Sefako Makgatho Health Sciences University, Pretoria, South Africa; 2 Division of Epidemiology and Biostatistics, School of Public Health, University of the Witwatersrand, Johannesburg, South Africa; 3 Department of Obstetrics and Gynaecology, School of Clinical Medicine, Faculty of Health Sciences, University of the Witwatersrand, Johannesburg, South Africa; 4 Department of Obstetrics and Gynaecology, Leratong Hospital, Krugersdorp, South Africa

**Keywords:** caregiver burden, family caregivers, ordinal logistic regression, predictors, schizophrenia, sociodemographic factors

## Abstract

**Background:**

Chronic mental illnesses such as schizophrenia affect patients’ functioning, making caregiving necessary although burdensome.

**Aim:**

This study aimed to determine caregiver burden and its sociodemographic determinants in family caregivers of patients with schizophrenia attending a Psychiatric Outpatient Department (POD).

**Setting:**

Tertiary hospital in Northern Pretoria, South Africa.

**Methods:**

In this cross-sectional study conducted over 3 months, 300 consecutive family caregivers who attended the POD were administered a 22-item Zarit Burden Interview (ZBI-22), which has a score of 0–88, with higher values indicating more burden. Their sociodemographic characteristics were ascertained. Linear and ordinal logistic regression analyses were performed to identify determinants or predictors of total and severe burdens, respectively.

**Results:**

Most caregivers were aged 46.0 ± 14 years, females (62%), parents (39%), of low-income status (93.7%), had secondary education (70%), resided with the patient (87%), and helped with all troublesome activities (95.3%). The median ZBI-22 score was 19.0 (interquartile range: 13.0–30.5). The determinants of both total and severe burdens were: caregiver age ≥ 50 years adjusted odds ratio (aOR): 2.55, confidence interval (CI): 1.49–4.36; residential area farther away from the hospital aOR: 1.76, CI: 1.3–2.99; increasing months of caregiving aOR: 1.0, CI: 1.001–1.009, *p* = 0.006; and not having another family member that needs care aOR: 0.43, CI: 0.24–0.78.

**Conclusion:**

Having mental healthcare facilities close to residential areas and assisting caregivers aged ≥ 50 years who have multiple family members who need care may alleviate the burden.

**Contribution:**

Predicting total and severe caregiver burdens contemporaneously is effective for identifying potential burden interventions.

## Introduction

Schizophrenia is a major psychiatric disorder with variable clinical presentation and chronic course, which are disabling to the patients.^1^ It constitutes a huge burden to the patient’s family members and the healthcare system.^2,3^ According to the World Health Organization, it affects 24 million or 1 in 300 persons (0.33%) worldwide.^4^ In South Africa, the exact prevalence of schizophrenia is unknown.^5^ However, the data from the 2019 global burden of schizophrenia shows that South African region has the following estimates: age-standardised prevalence 220–260, incidence 13–15, and disability-adjusted life years (DALYs) 140–160 rates per 100 000.^6^ The same data show an increase in the burden of schizophrenia, and that the global estimates with 95% uncertainty interval were age-standardised prevalence 287.41 (246.16–330.88), incidence 16.31 (13.80–19.42) and DALYs 184.15 (134.32–234.54) rates per 100 000. Schizophrenia is a major cost driver of mental healthcare and results in a huge fiscal loss of approximately $56 707 per life year lived with schizophrenia in the US.^3,7^ The disorder has a genetic predisposition and is associated with environmental factors such as substance use, and may be precipitated or aggravated by poor socioeconomic conditions.^8,9,10^ It is a major mental health issue in settings where substance use is prevalent.^9^

Patients with schizophrenia require additional support at home from their family members, and this is often on a long-term basis because of the impact of the disease on patients’ functioning. This includes impairment and distress in the personal, family, occupational, social, and other crucial areas of activities of daily living.^4^ These limitations are the functional implications of schizophrenia that result in long-term care. Long-term care is a range of services and supports that an individual requires for personal care needs.^11,12^ The family member who provides unpaid care to the patient is called an informal caregiver.^13^ This care can be burdensome to the caregiver. According to Zarit and colleagues, perceived burden is ‘one’s subjective belief that current and future resources are insufficient to meet role demands’.^14^ The three major attributes that explain the development of the burden are self-perception by the caregiver, multifaceted stressors, and the occurrence of these over a period.^15^ The interaction of these attributes results in primary stressors that the caregiver may or may not be able to cope with (stress-coping perspective) and may generate an impact on other aspects of the caregiver’s life such as employment and family, to give rise to secondary stressors.^16^ For instance, caring for patients with schizophrenia may change the family dynamics of the caregivers resulting in dysfunctional households including caregivers’ difficulty in coping with their spouses and children and these family members often feel alienated by the caregiver.^15^

Stress-coping-burden perspective may be influenced by the sociodemographic context of the caregiver. The sociodemographic context refers to sociodemographic factors peculiar to an environment or setting. For instance, a previous study in South Africa showed that a low educational attainment was associated with poor quality of life and a high caregiver burden, while a high household income had the opposite effect.^9^ These findings resonate with a report from Cambodia where paid employment was associated with a high quality of life while low-income households and chronic diseases were associated with a poor quality of life among family caregivers.^17^ As a result, knowledge of the burden, its severity,^18^ and the sociodemographic determinants may result in the identification of interventions to assist the affected caregivers and their families. The sociodemographic determinants in this context are sociodemographic variables that may predict caregiver burden. Implementation of the interventions may also have an impact on the patient’s care. Therefore, it is pertinent to investigate the sociodemographic determinants (predictors) of both total and severe caregiver burdens (using analysis methods such as linear and ordinal logistic regression models, respectively). This is crucial given that caregiver burden has a spectrum and it may not be practically possible to investigate all the spectra based on omnibus study on total burden score. Moreover, severe burden may be more debilitating than non-severe types and require specific research to be conducted on it, to aid its prevention. Thus, we utilised linear regression for total burden score and utilised ordinal logistic regression for the spectrum of none to low, mild to moderate, moderate to severe, and severe burdens. Although the most important thing is to identify and fill the gaps in the literature, this approach will pick out sociodemographic variables that will predict both total and severe burdens in the same group of research participants. The aim of this study, therefore, was to ascertain the caregiver burden and its sociodemographic determinants on family caregivers of patients with schizophrenia attending the Psychiatric Outpatient Department (POD) in a tertiary hospital in South Africa.

## Methods

### Study design, setting, and duration

This was a cross-sectional study conducted in the POD of a tertiary hospital in northern Pretoria, Gauteng province, South Africa from 01 June 2022 to 31 August 2022.

### Study population

The study population was the family caregivers of patients with schizophrenia attending a tertiary hospital in northern Pretoria, South Africa, that provided at least weekly care to the patients. In the study setting, patients with schizophrenia who attend the outpatient psychiatry clinic are usually accompanied by their family caregivers during hospital visits. At other times, these family caregivers visit the clinic unaccompanied to collect chronic medications on behalf of the patients. Our sample was drawn from these family caregivers. All eligible family caregivers were offered the opportunity to participate in the study.

### Selection criteria

#### Inclusion criteria

The following participants were included: (1) family members who were caregivers of patients with schizophrenia attending the POD at the study setting during the period of data collection, (2) participants aged 18 years and above, and (3) caregivers who provided regular care (which may be physical, psychological, and/or financial) to the patients with schizophrenia. For this study, regular care was defined as frequency of care of at least once a week (personal communication with Professor Stephen H Zarit, 2021).

#### Exclusion criteria

We excluded caregivers who were paid for providing care to the recipient.

### Sampling

#### Sample size

Based on a 5% margin of error, 99% confidence interval, a population size of 540 family caregivers in 3 months obtained from the POD records of visits, and a response distribution rate of 50%, we obtained a sample size of 298 (approximately 300), using the Raosoft sample size software.^19^ This sample size was sufficient to ensure an event per variable of at least 10 for the regression analysis (i.e. the number of observations were always 10 times more than the number of predictors included in each regression model).

#### Sampling method

The family caregivers of patients with schizophrenia who attended the POD during the study period were informed to participate in the study. The exclusion and inclusion criteria were applied. A convenience sampling method was used. All eligible, consented, and consecutively identified caregivers attending the POD were included in the study until the sample size was attained.

### Data collection

#### Caregiver burden assessment tool

According to Zarit and colleagues, perceived burden is ‘One’s subjective belief that current and future resources are insufficient to meet role demands’.^14^ One of the most popular instruments widely used to measure caregiver burden is the 22-item Zarit Burden Interview (ZBI-22) https://wai.wisc.edu/wp-content/uploads/sites/1129/2021/11/Zarit-Caregiver-Burden-Assessment-Instruments.pdf. It assesses the burden of caring for patients such as those with mental illnesses including schizophrenia.^20^ The ZBI scale assesses the impact of caregiving on the life of the caregiver. In terms of dimensionality, it explores personal and role strains^21^ as well as other domains including physical, mental, social, and financial impacts the patient’s care has on the caregiver.^22,23,24,25^ The questionnaire may be self- or interviewer-administered.^26^

The responses to the ZBI-22 are rated on a Likert scale ranging from 0 (never) to 4 (almost always) and the total score ranges from 0 to 88. Higher scores imply that there is a higher level of burden experienced by the caregiver. The total score may be categorised according to the severity of the burden into 0–20 (none to low), 21–40 (mild to moderate), 41–60 (moderate to severe burden), and 61–88 (severe).^18^ The ZBI-22 has a Cronbach’s alpha (or coefficient alpha) of 0.93 and an intra-class correlation coefficient for test-retest reliability of 0.89.^27^ These parameters are the most suitable and commonly used measures for assessing internal consistency and reliability.^28^ The instrument has been used in different parts of the world including Africa, Asia, and South America to assess caregiver burden. It has been validated among Africans^29^ and other investigators in South Africa have used it to assess caregiver burden.^30^ The ZBI-22 has also been validated among informal caregivers of patients with schizophrenia and found to be an appropriate tool by experts.^31,32,33^ According to other investigators, the ZBI-22 is the gold standard for assessing family caregiver burden in patients with schizophrenia.^34^

#### Data collection process

**FIGURE 1:**
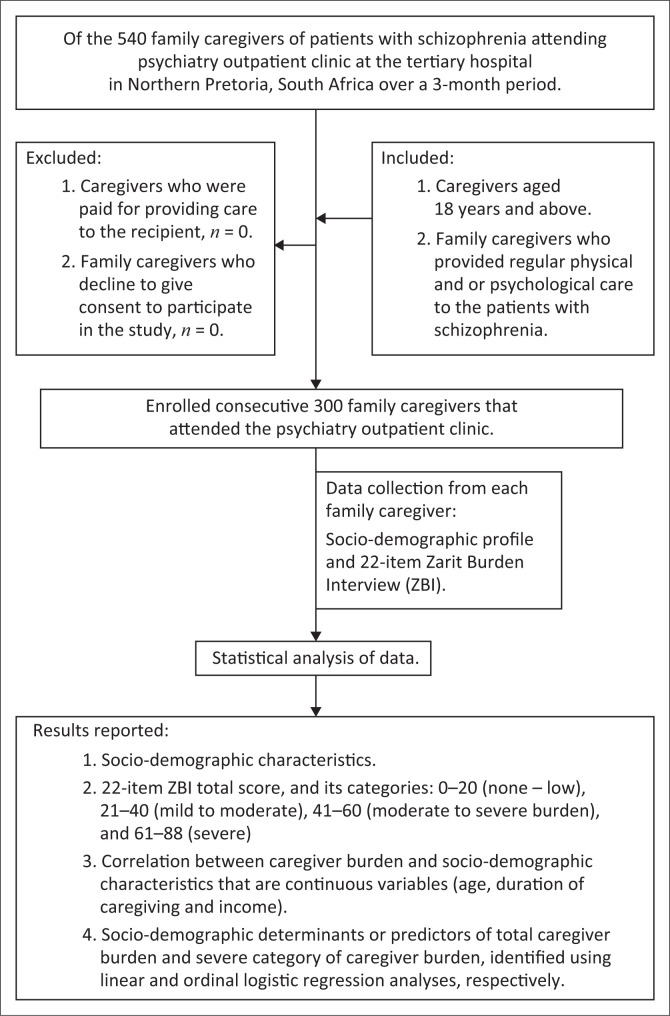
Overview of the study process.

Again, the family caregivers of patients with schizophrenia who attended the POD during the study period were informed to participate in the study. The exclusion and inclusion criteria were applied. The ZBI-22 questionnaire^27,34^ was administered to each participant by the principal investigator through one-to-one interviewing. In addition, the caregivers provided details of their sociodemographic information (Figure 1). Both the ZBI and the sociodemographic data were entered into a data collection sheet. The data were subsequently captured by the principal investigator using SPSS version 27.0 (IBM, Armonk, NY, USA) based on personal preference.

### Statistical analysis of data

Data were analysed using STATA version 17 (StataCorp, Texas, USA). Categorical variables were expressed as frequency and percentages (Table 1, Table 2, and Table 3). The prevalence of the four categories of severity of caregiver burden (none to low [0–20]; mild to moderate [21–40]; moderate to severe [41–60]; severe [61–88]) was also expressed as frequency and percentage with its 95% confidence interval (CI). The normality of the continuous variables was assessed with multiple measures including the skewness-kurtosis test. Normally distributed data were expressed as mean and standard deviation. Skewed data were expressed as median and interquartile range (IQR). The correlation between continuous variables among sociodemographic characteristics and total caregiver burden score was assessed using Spearman rank correlation. Missing data were excluded from the calculation of *p*-values.

**TABLE 1:** Caregiver sociodemographic characteristics (*N* = 300).

Variable	Value	
	Frequency	%
**Residence**		
Northern Gauteng (same area as the study setting)	135	45.00
Other areas in Gauteng	128	42.70
Outside Gauteng	20	6.70
Missing data	17	5.70
**Age (years)**	-	-
< 50	174	58.00
≥ 50	125	41.67
Missing data	1	0.30
**Gender**		
Female	186	62.00
Male	114	38.00
**Marital status**		
Single	207	69.00
Married	70	23.30
Widowed	11	3.70
Divorced	10	3.30
Missing data	2	0.70
**Relationship with patient**		
Parent	117	39.00
Spouse	12	4.00
Child	49	16.30
Others	121	40.30
Missing data	1	0.30
**Race**		
Black people	295	98.30
Asian people	0	0.00
Caucasian people	0	0.00
Mixed race people	4	1.30
Missing data	1	0.30
**Religion**		
Christianity	293	97.70
Islam	2	0.70
Others	4	1.30
No religion	1	0.30
**Highest level of education**		
Primary	29	9.70
Secondary	210	70.00
Tertiary	35	11.70
None	25	8.30
Missing data	1	0.30
**Employment status**		
Unemployed	243	81.00
Employed	56	18.70
Missing data	1	0.30
**Level of income as classified by caregiver**		
Low	281	93.70
Moderate	18	06.00
High	0	0.00
Missing data	1	0.30
**Occasional inability to feed while hungry because of a lack of food and money?**		
Yes	18	06.00
No	282	94.00
**Co-morbidity in the caregiver**		
None	130	43.30
Psychiatry	8	2.70
Medical	112	37.30
Surgical/Gynaecological	4	1.30
Others	40	13.30
Missing data	6	2.00
**Do you smoke cigarette?**		
Yes	50	16.70
No	250	83.30
**Do you drink alcohol?**		
Yes	58	19.30
No	242	80.70
**Do you use any other substance?**		
Yes	1	0.30
No	299	99.70

Note: Age: Mean ± s.d. = 46.0 ± 14.9; Monthly income (ZAR): Median = 1900; Inter quartile range: 1900–2250.

s.d., standard deviation.

**TABLE 2:** Caregiving socio-demographic characteristics (*N* = 300).

Variable	Value	
	Frequency	%
**Do you have any other family member who requires care?**		
Yes	75.0	25.0
No	22.0	74.7
Missing data	1.0	0.3
**Are you living with the patient?**		
Yes	261.0	87.0
No	39.0	13.0
**Frequency of care per week**		
Everyday	258.0	86.0
Twice a week	14.0	4.7
More than twice a week	3.0	1.0
Missing data	25.0	8.3
**Have any of your family members had COVID-19?**		
Yes	24.0	8.0
No	275.0	91.7
Missing data	1.0	0.3
**Do you help the patient with all activities that he or she has trouble doing?**		
Yes	286.0	95.3
No	14.0	4.7
**Type of care provided to the patient by caregiver**		
Physical, psychological and financial support	194.0	64.7
Physical and psychological support	43.0	14.3
Other combinations of care including managing patient’s finances	63.0	21
**Medium of providing care to patient**		
Physically available	202.0	67.3
Phone calls	12.0	4.0
Other electronic media	1.0	0.3
Others	1.0	0.3
Both physical and phone calls	83.0	27.7
Missing data	1.0	0.03
**Weeks of break (leave) taken (within the last one year) from providing care to the patient**		
None	262.0	87.3
Less than one week	22.0	7.3
One to four weeks	6.0	2.0
More than four weeks	5.0	1.7
Missing data	5.0	1.7

Note: Duration of months of caregiving to patient: Median = 24; Inter quartile range = 12–48.

**TABLE 3:** Prevalence of severity of caregiver burden.

Severity of caregiver burden using Zarit burden interview	Frequency	%	95% CI
None to low (0–20)	165	55.00	49.30–60.6
Mild to moderate (21–40)	92	30.67	25.70–36.1
Moderate to severe (41–60)	36	12.00	8.80–16.2
Severe (61–88)	7	2.33	1.10–4.8

**Total**	**300**	**100.00**	**-**

Note: The median (and interquartile range) of the total caregiver burden score was 19.00 (13.00–30.50).

CI, confidence interval.

Simple and multiple linear regression were further conducted with the total burden of care score as the outcome or dependent variable while the sociodemographic characteristics were the explanatory variables. Variables with univariable *p* < 0.2 were selected for the multivariable linear regression.^35,36^ However, the gender of the caregiver, the relationship of the caregiver with the patient, and self-reported income per month were chosen a priori based on theoretical and pragmatic knowledge. The multivariable linear model was built using the backward elimination technique.^37^ To increase the efficiency of the analysis, the four categories of caregiver burdens were taken as an ordinal or ordered variable^38^ and a proportional odds model (POM) of ordinal logistic regression was conducted.^39,40,41^ Similarly, univariable and multivariable ordinal regression was built as previously described for linear regression. The ologit command in STATA was used, and variables that did not violate the assumptions of POM or rule of parallel odds based on Brant’s test and ‘omodel’ test were utilised for the modelling. Multicollinearity was assessed among the variables using the variance inflation factor and values less than 10 suggest no collinearity.^42,43^ Two-tailed test of the hypothesis was assumed, and a *p* < 0.05 was taken as a statistically significant level. The goodness-of-fit test (Hosmer-Lemeshow, Pulkstenis-Robinson, and Lipsitz chi-square tests) was calculated, and a *p* > 0.05 showed model fit.

### Ethical considerations

The study protocol was reviewed and approved by the Sefako Makgatho Health Sciences University (SMU) Research and Ethics Committee, reference SMUREC/M/01/2022:PG. Each participant gave informed consent before participating in the study after the nature of the research had been fully explained. The permission to re-use the ZBI-22 was obtained from the publisher, Oxford University Press. To ensure confidentiality, the data collected from participants did not have personal identification of the caregiver such as the name. Rather, a study code was used to de-identify the participants. The collected data were stored in a lockable cupboard with access control.

## Results

### Sociodemographic characteristics

Table 1 and Table 2 show the sociodemographic characteristics of the caregivers (*N* = 300), and their mean age was 46.0 ± 14 years. Most caregivers were females (62%), parents (39%), of low-income status (93.7%), had secondary education (70%), resided with the patient (87%), and helped with all troublesome activities (95.3%).

### Caregiver burden score

The Cronbach’s alpha of the 22-item ZBI in our study was 0.86. The minimum and maximum total caregiver burden scores were 0 and 75, respectively, while the median was 19.00 (IQR: 13.0–30.8). Among all participants, ZBI items 2, 4–6, 9–14, and 16–19 had a median score of zero out of four. Items 15 and 21 had the highest median scores of 3 (IQR: 2–4) and 3 (IQR: 2–3), respectively. Other items had median scores ranging from 1 to 2. Table 3 shows the prevalence of severity of caregiver burden. The prevalence of different categories of caregiver burden decreased with increasing severity of burden: none to low 55%, mild to moderate 30.67%, moderate to severe 12%, and severe 2.33%.

### Correlation between sociodemographic continuous variables and total caregiver burden

There was a positive moderate correlation between age (*r* = 0.4, *p* < 0.001), duration of caregiving (*r* = 0.37, *p* < 0.001) and the total caregiver burden. On the other hand, there was a non-statistically significant negative correlation between the monthly income and total caregiver burden (*r* = −0.05, *p* = 0.512).

### Determinants of total caregiver burden

Table 4 shows that at multivariable linear regression (after adjusting for covariates), the statistically significant explanatory variables were the total duration of months of caregiving, having another family member that requires care, use of alcohol, educational status, marital status, age, residence, and smoking. The *R*^2^ of these predictors during univariable analysis were: total duration of months of caregiving 0.1232, age 0.1229, marital status 0.1061, having another family member who had needs for care 0.0620, educational status 0.0475, alcohol ingestion 0.0424, place of residence 0.0356, and smoking 0.0309.

**TABLE 4:** Simple and multiple linear regression of the predictors of total caregiver burden score.

Variable	Univariable			Multivariable		
	Crude *β*	95% CI	*p*	Adjusted *β*	95% CI	*p*
**Duration of months of caregiving**	0.07	0.05 to 0.09	< 0.001	0.05	0.02–0.07	0.002*
< 12	0.00	Reference	Reference	-	-	-
12–23	8.29	0.73–15.85	0.032	-	-	-
24–35	7.41	0.02–14.80	0.049	-	-	-
36–47	8.99	0.98–17.01	0.028	-	-	-
48–59	15.42	6.00–24.83	0.001	-	-	-
≥ 60	19.99	12.48–27.49	< 0.001	-	-	-
**Do you have another family member that requires your care?**						
Yes	0.00	Reference	Reference	0.00	Reference	Reference
No	−7.99	−11.55 to −4.44	< 0.001	−6.62	−11.07 to −2.16	0.004*
**Does caregiver drink alcohol?**						
Yes	0.00	Reference	Reference	0.00	Reference	Reference
No	7.25	3.32–11.18	< 0.001	6.25	0.49–12.01	0.034*
**Monthly income**	0.0004	−0.00003 to 0.0009	0.065	0.00035	−0.00012 to 0.00082	0.140
**Highest level of education of the caregiver**						
None	0.00	Reference	Reference	0.00	Reference	Reference
Primary	−8.63	−15.94 to −1.31	0.021	−5.05	−12.35 to 2.25	0.174
Secondary	−10.95	−16.63 to −5.28	< 0.001	−7.26	−13.59 to −0.93	0.025*
Tertiary	−8.98	−16.01 to −1.96	0.012	−11.54	−20.95 to −2.14	0.016*
**Gender of caregiver**						
Female	0.00	Reference	Reference	0.00	Reference	Reference
Male	−7.30	−10.45 to −4.14	< 0.001	−1.83	−5.90 to 2.24	0.376
**Relationship with the patient**						
Others	0.00	Reference	Reference	0.00	Reference	Reference
Parent	8.12	4.67 to 11.58	< 0.001	−4.02	−8.61 to 0.57	0.085
Spouse	4.35	−3.72 to 12.41	0.290	1.92	−7.36 to 11.19	0.684
Child	3.76	−0.75 to 8.27	0.102	−2.24	−7.98 to 3.50	0.442
**Marital status**						
Single	0.00	Reference	Reference	0.00	Reference	Reference
Married	6.44	2.83–10.05	0.001	1.51	−2.80 to 5.82	0.491
Widowed	11.71	3.62–19.79	0.005	6.35	−3.13 to 15.83	0.188
Divorced	19.35	10.89–27.81	< 0.001	14.72	4.92 to 24.52	0.003*
**Age (years)**	0.35	0.25–0.45	< 0.001	-	-	-
< 50	0.00	Reference	Reference	0.00	Reference	Reference
≥ 50	9.89	6.87–12.91	< 0.001	5.04	0.46 to 9.61	0.031*
< 30	0.00	Reference	Reference	-	-	-
30–39	−1.18	−6.02 to 3.66	0.633	-	-	-
40–49	1.71	−2.98 to 6.40	0.474	-	-	-
50–59	8.78	4.10–13.45	< 0.001	-	-	-
60–69	8.04	2.74–13.33	0.003	-	-	-
≥ 70	18.79	12.14–25.43	< 0.001	-	-	-
**Residence**						
Northern Gauteng (same area as study setting)	0.00	Reference	Reference	0.00	Reference	Reference
Other areas in Gauteng	−0.34	−3.69 to 3.01	0.842	1.82	−2.06 to 5.13	0.400
Outside Gauteng	10.09	3.59–16.59	0.002	3.79	1.44 to 16.41	0.020*
**Does caregiver smoke cigarette?**						
Yes	0.00	Reference	Reference	0.00	Reference	Reference
No	6.56	2.37–10.74	0.002	−6.28	−12.44 to −0.12	0.046*
**Race**						
Black people	0.00	Reference	Reference	-	-	-
Mixed race people	0.37	−13.47 to 14.22	0.958	-	-	-
**Religion**						
Christianity	0.00	Reference	Reference	-	-	-
Others	8.82	−1.63 to 19.27	0.098	-	-	-
**Employment status**						
Unemployed	0.00	Reference	Reference	-	-	-
Employed	2.26	−1.80 to 6.32	0.274	-	-	-
**Income class**						
Low	0.00	Reference	Reference	-	-	-
Moderate	−0.28	−6.95 to 6.40	0.935	-	-	-
**Occasionally no food while hungry**						
Yes	0.00	Reference	Reference	-	-	-
No	−14.01	−20.49 to −7.53	< 0.001	-	-	-
**Does caregiver use any other substance?**						
Yes	0.00	Reference	Reference	-	-	-
No	12.42	−15.04 to 39.89	0.374	-	-	-
**COVID-19 diagnosis in a family member**						
Yes	0.00	Reference	Reference	-	-	-
No	−8.80	−14.57 to −3.04	0.003	-	-	-
**Living with patient**						
Yes	0.00	Reference	Reference	-	-	-
No	−9.16	−13.76 to −4.57	< 0.001	-	-	-
**Type of comorbidity in the caregiver**						
None	0.00	Reference	Reference	-	-	-
Psychiatry	4.03	−5.86 to 13.93	0.423	-	-	-
Medical	1.95	−1.56 to 5.45	0.275	-	-	-
Surgical / Gynaecological	−5.22	−19.00 to 8.57	0.457	-	-	-
Others	6.33	1.42 to 11.25	0.012	-	-	-
**Frequency of care per week**	−0.80	−4.46 to 2.87	0.669	-	-	-
**Frequency of care per week**						
Everyday	0.00	Reference	Reference	-	-	-
Twice a week	−1.11	−8.61 to 6.39	0.771	-	-	-
More than twice a week	−5.59	−21.46 to 10.29	0.489	-	-	-

Note: *R*^2^ = 42.16%. Multivariable analysis columns contain variables that were not eliminated during backward elimination regression analysis and those chosen a priori.

CI, confidence interval.

* , Statistical significance, *p* < 0.5.

In Table 4, the linear regression coefficient (*β*) shows the expected change in the dependent variable that is caused by one unit change in the predictor variable. For every 1-month increase in the duration of caregiving, the total caregiver burden increased by 5% (adjusted *β* [a*β*] = 0.05, 95% CI: 0.02–0.07, *p* = 0.002). The caregiver burden score of participants who did not have another family member who requires attention was about 6.6 lesser as compared to participants with another family member who needed care (a*β* = −6.62, 95% CI: −11.07 to −2.16, *p* = 0.004). Furthermore, participants who do not drink alcohol had on average about 6.6 extra burden of care score as compared to those who drank alcohol (a*β* = 6.62, 95% CI: −0.49 to 12.01, *p* = 0.034). The burden of care score generally decreased with increasing level of education. Participants who were 50 years and older had on average about 5.04 extra burden of care score as compared to participants who were younger than 50 years (a*β* = 5.04, 95% CI: 0.46 to 9.61, *p* = 0.031). Participants living outside Gauteng had on average about 3.8 extra burden score compared to participants living in Northern Gauteng. However, there was no statistically significant difference in burden score between participants living in other parts of Gauteng and those living in Northern Gauteng. In addition, a participant who was a divorcee had a higher total burden of care score of 14.72 as compared to the burden among single participants. A habit of not smoking reduces the total burden of caregiving by 6.28 in comparison to smoking. The variance inflation factor of all the covariates was less than 2, suggesting that there was no collinearity among the variables.

### Determinants of severe caregiver burden

After multivariable ordinal logistic regression modelling of the predictors of severe burden of care, the explanatory variables that were statistically significant were having another family member that requires care, gender, age category (with 50 years as threshold), residence, and duration of months of caregiving (Table 5). Notably, reporting results of an odds ratio as a percentage change in the outcome (dependent) variable given an exposure to a risk factor (explanatory variable) is valuable. The formula for the percentage change is odds ratio minus 1 multiplied by 100.^44^ There was a 57% lesser likelihood of having a severe burden of care among participants with no other family member with needs of care as compared to participants who had another family member with needs of care (aOR: 0.43, 95% CI: 0.24–0.78, *p* = 0.006). Likewise, there were 51% lesser odds of having a severe burden of care among males as compared to females (aOR: 0.49, 95% CI: 0.28–0.87, *p* = 0.015). The likelihood of having a severe burden of care among older participants who were 50 years and older was about 2.6 times the odds of having a severe burden of care among participants who were younger than 50 years (aOR: 2.55, 95% CI: 1.49–4.36, *p* = 0.001). The odds of having a severe burden of care increases with increasing distance of the residence from the study site. Thus, the odds of having a severe burden of care were about 76% higher among participants living in other areas of Gauteng as compared to those living in Northern Gauteng (aOR: 1.76, 95% CI: 1.03–2.99, *p* = 0.038). The likelihood of having a severe burden of care increases by 0.5% for every additional 1 month of caring for the patients (aOR: 1.005, 95% CI: 1.001–1.009, *p* = 0.006). The self-reported income class of the participants did not influence the risk of having a severe burden of care even after multivariable ordinal regression modelling (POM). There was no violation of the POM based on the result of Brant’s test (X^2^ = 13.71, *p* = 0.472) and the omodel result (X = 5.57, *p* = 0.936). The goodness of fit test showed that Hosmer-Lemeshow (*p* = 0.378), Pulkstenis-Robison (*p* = 0.981), and Lipsitz (*p* = 0.063) tests all had *p* > 0.05.

**TABLE 5:** Univariable and multivariable ordinal logistic regression of factors affecting burden of care of participants.

Variable	Univariable			Multivariable			
	Crude odds ratio	95% CI	*p*	Adjusted odds ratio	95% CI		*p*
					Lower	Upper	
**Do you have another family member who requires your care?**							
Yes	1.00	Reference	Reference	1.000	Reference	Reference	Reference
No	0.35	0.21–0.58	< 0.001	0.430	0.240	0.780	0.006*
**Gender of caregiver**							
Female	1.00	Reference	Reference	1.000	Reference	Reference	Reference
Male	0.33	0.21–0.54	< 0.001	0.490	0.280	0.870	0.015*
**Age (years)**							
< 50	1.00	Reference	Reference	1.000	Reference	Reference	Reference
≥ 50	3.58	2.26–5.67	< 0.001	2.550	1.490	4.360	0.001*
**Duration of months of caregiving**	1.01	1.00–1.01	< 0.001	1.005	1.001	1.009	0.006*
**Residence**							
Northern Gauteng (same area as study setting)	1.00	Reference	Reference	1.000	Reference	Reference	Reference
Other areas in Gauteng	1.33	0.83–2.14	0.237	1.760	1.030	2.990	0.038*
Outside Gauteng	3.71	1.50–9.22	0.005	1.830	0.670	5.010	0.242
**Income class**							
Low	1.00	Reference	Reference	1.000	Reference	Reference	Reference
Moderate	0.77	0.30–2.00	0.592	0.590	0.210	1.610	0.301
**Occasionally no food while hungry**							
Yes	1.00	Reference	Reference	-	-	-	-
No	0.18	0.07–0.43	< 0.001	-	-	-	-
**Education level**							
None	1.00	Reference	Reference	-	-	-	-
Primary	0.30	0.11–0.84	0.021	-	-	-	-
Secondary	0.28	0.13–0.60	0.001	-	-	-	-
Tertiary	0.35	0.13–0.93	0.035	-	-	-	-
**Religion**							
Christianity	1.00	Reference	Reference	-	-	-	-
Others	3.06	0.76–12.32	0.116	-	-	-	-
**Marital Status**							
Single	1.00	Reference	Reference	-	-	-	-
Married	2.40	1.44–4.03	0.001	-	-	-	-
Widowed	3.34	1.06–10.55	0.040	-	-	-	-
Divorced	9.84	3.09–31.37	< 0.001	-	-	-	-
**Employment Status**							
Unemployed	1.00	Reference	Reference	-	-	-	-
Employed	1.25	0.71–2.19	0.435	-	-	-	-
**Frequency of care per week**	0.68	0.38–1.23	0.202	-	-	-	-
**Frequency of care per week**							
Everyday	1.00	Reference	Reference	-	-	-	-
Twice a week	0.49	0.15–1.61	0.241	-	-	-	-
More than twice a week	0.48	0.05–4.97	0.539	-	-	-	-

Note: Multivariable analysis columns contain variables that were not eliminated during backward elimination regression analysis and those chosen *a priori*.

CI, confidence interval.

* , Statistically significant level: *p* < 0.05.

## Discussion

### Main findings

The median ZBI-22 score was 19.0 (IQR: 13.0–30.5). The explanatory variables that predicted total and severe caregiver burdens in multivariable linear and ordinal logistic regression analyses, respectively, were four: age ≥ 50 years, total duration of months of caregiving, residence farther away from the hospital, and having another family member that needs care.

### Interpretation

The median (and IQR) total caregiver burden score was 19.00 (13.00–30.50). Our finding is comparable to the ZBI-22 median score of 11 found by Martyns-Yelllowe^45^ and a mean score of 25.8 ± 7.4 reported by Khalil et al.^46^ However, a mean score as high as 51.7 ± 18.2 and 56.1 ± 17.6 have been reported by Shamsaei et al.^2^ and Yerriah et al.,^9^ respectively. In our study, the distribution of severity of burden among the caregivers was ‘no to low’ 55%, ‘mild to moderate’ 30.7%, ‘moderate to severe’ 12%, and ‘severe’ burden 2.3%. Shamseai et al., in their study, reported that 7.6% of the caregivers had ‘no to low’, 23.5% ‘mild to moderate’, 41.8% ‘moderate to severe’, and 27.1% ‘severe’ burden.^2^ The frequency of burden in our study decreased with increasing severity of the burden category. This is important as only a few participants had severe burden, which may signify that most of the caregivers have good support and coping mechanisms. However, it is difficult to comment further on the possible support and coping mechanisms because of the absence of supporting data as the study’s design did not include data collection on these variables. Although more than half of our participants reported none to low burden, targeted interventions to increase the prevalence of ‘none to low’ burden scores among the caregivers are of essence. This is because a low burden is associated with a high quality of life among caregivers of patients with schizophrenia in South Africa.^9^ It has been reported from South Africa that among family caregivers of patients with schizophrenia and comorbid substance use, little or no burden occurred in 4 (3.9%) while 81 (80.2%) had high or severe caregiver burden.^9^ Differences in patients’ profiles, and caregivers’ personal and caregiving characteristics may account for the variations in the ZBI-22 scores found in various studies. For instance, substance use among patients with schizophrenia is associated with a high caregiver burden.^9^

The four explanatory variables that predicted total and severe caregiver burdens in multivariable linear and ordinal logistic regression analyses, respectively, were age ≥ 50 years, total duration of months of caregiving, residence farther away from the hospital, and having another family member that needs care. Arguably, increasing age and duration of caregiving are associated with increasing responsibility, which may be a source of stress that culminates in burden. For instance, other authors have reported a statistically significant positive correlation between caregiver age and burden.^47^ A counterargument however is that the burden may be reduced if there is skill acquisition and adaptation with increasing age and duration of caregiving. Nonetheless, other authors have shown that a decreased number of contact hours between patients with schizophrenia and their caregivers reduced the caregivers’ distress.^48^ Furthermore, caregivers who live farther away from the hospital are at high risk of travelling more distance to arrive at the hospital to receive services and may incur a high cost of transportation, which may cause financial strain. This is of particular importance in the study setting given that most of the caregivers are of low-income status. Additionally, the multifaceted strains incurred from caring for more than one patient may explain why having another family member who needs care is a determinant of caregiver burden. In previous studies involving family caregivers of patients with schizophrenia, the patient’s age, monthly income, and functioning as well as the caregiver’s gender, level of education, occupation, and the duration of time the caregiver spent with the patient in the same house were predictors of caregiver burden.^49,50^ Using the Family Burden Interview Schedule (FBIS), Yu et al. found that gender, education, and having an additional dependent were predictive of caregiver burden in family members of patients with schizophrenia.^51^ The variation between the type of predictors identified in our study and those found in other studies may be because of differences in attributes comprising sociodemographic characteristics, strains encountered by the caregivers, and personal perceptions, as these may influence the development and severity of the burden.^15^ Furthermore, the type of tool used to measure caregiver burden, and whether or not both total and severe burdens were contemporaneously assessed may explain the variation in findings from different studies.

The *R*^2^ (coefficient of multiple determination or coefficient of determination) of the multivariable linear regression model was 42.16% (Table 4). This implies that 42.16% of the variance in the dependent variable (total caregiver burden) is explained by the independent variables. Human behaviour is generally difficult to predict and studies about them generally have an *R*-squared of less than 50%.^52^

### Strengths and limitations

This is one of the few studies on caregiver burden of family caregivers of patients with schizophrenia conducted during the era of coronavirus disease 2019 (COVID-19). To the best of our knowledge, it is the first study that identified the determinants of both total and severe caregiver burdens associated with schizophrenia (contemporaneously in the same group of caregivers). This approach was undertaken to improve the efficiency of predicting both total and severe burdens contemporaneously. Furthermore, we used an instrument that has domains that comprehensively assess caregiver burden including the stressors such as patient’s behaviour as in item 4 in ZBI-22.

The main limitation of our study is that the sociodemographic profile of caregivers may be different in various settings. However, the findings of this study may apply to settings with similar sociodemographic profiles as ours. In addition, the sampling method was a convenience sampling, which may predispose to selection bias. To prevent selection bias, all eligible and consecutively identified caregivers who attended the POD during the period of data collection were included in the study until the sample size was attained. No caregiver declined to participate in the study. Furthermore, there is a lack of robust data on cultural determinants of caregiver burden. Consequently, culturally guided interventions to mitigate the burden of care on family caregivers of patients with schizophrenia require further investigation. This is therefore a future research agenda. Furthermore, we wish to acknowledge that the interpretation and/or expression of the burden may be influenced by the disabilities in the caregivers themselves. Although the chronic illnesses of the participants were ascertained, it is proposed that future studies should address this limitation. Moreover, we did not compare the burden of care across family caregivers of patients with different types of chronic medical conditions. Future studies that will provide this information are required.

### Recommendations

A major implication of our study is that it points to recommendations on how to reduce caregiver burden. Firstly, the severity of caregiver burden worsened with increasing duration of months of care. This may suggest that many patients required prolonged long-term care in their activities of daily living because of inadequate or difficult symptom control. Therefore, we recommend regular patient follow-up (as clinically indicated) with easy access to healthcare services. Symptom remission will also allow the caregiver to engage in support groups and skills training/acquisition activities, the latter being crucial since the majority were unemployed.

Secondly, family caregivers aged 50 years and above were identified to be at high risk of having an increased burden; therefore, this age group should be evaluated periodically to determine the severity of their burden. Those with severe burdens should be offered individualised support such as the opportunity for the patients with schizophrenia to be considered for placement in an institution. This also applies to caregivers who have more than one family member that needs care. Of note, accepting placement can be a very difficult decision for many families. Therefore, other alternative interventions should be explored and offered, given that placement shifts some but does not relieve all associated family burdens.^53^ For every caregiver, however, individualised intervention is crucial because the stressors resulting in the experienced burden may be different. These interventions that may be offered to the caregiver by healthcare professionals to reduce burden include providing education and information, assisting with managing the caregiver’s health conditions, and referring them to other multidisciplinary teams for assistance. Other interventions include to encourage the caregiver to improve self-care and maintain good health, make and execute plans to mitigate identified stressors, use supportive technology where possible, as well as consider respite care services.^53,54^

Thirdly, improving transportation services may alleviate the burden among caregivers who reside far away from the hospital. This includes improving both patient-paid public transport systems, and making available efficient ambulance services that will collect patients and caregivers at specific places in the community and convey them to the hospital to receive medical assistance. Currently, in South Africa, patients are transported from their homes to the hospitals only if they have emergency medical conditions and not stable chronic illnesses.

## Conclusion

Family caregivers who care for patients with schizophrenia are at risk of developing a caregiver burden. Age, residential area, duration of caregiving, and having another family member who needs care are determinants of both total and severe caregiver burden. Having mental healthcare facilities close to residential areas, and assisting families aged ≥ 50 years with more than one member that needs long-term care are interventions that may reduce burden. An encounter with these family caregivers for their healthcare needs should be an opportunity for the practitioners to discuss and initiate appropriate interventions to reduce the burden.
